# Differential impacts of fat and muscle mass on cardiovascular and non‐cardiovascular mortality in individuals with type 2 diabetes

**DOI:** 10.1002/jcsm.13542

**Published:** 2024-07-12

**Authors:** Jie Guo, Yuxia Wei, Emerald G. Heiland, Anna Marseglia

**Affiliations:** ^1^ Department of Nutrition and Health China Agricultural University Beijing China; ^2^ Aging Research Center, Department of Neurobiology, Care Sciences and Society Karolinska Institutet Solna Sweden; ^3^ Institute of Environmental Medicine Karolinska Institutet Solna Sweden; ^4^ Medical Epidemiology, Department of Surgical Sciences Uppsala University Uppsala Sweden; ^5^ Department of Physical Activity and Health The Swedish School of Sport and Health Sciences (GIH) Stockholm Sweden; ^6^ Division of Clinical Geriatrics, Center for Alzheimer Research, Department of Neurobiology, Care Sciences and Society Karolinska Institutet Huddinge Sweden

**Keywords:** body composition, dual‐energy X‐ray absorptiometry, mortality, type 2 diabetes

## Abstract

**Background:**

The distribution of fat and muscle mass in different regions of the body can reflect different pathways to mortality in individuals with diabetes. Therefore, we investigated the associations between whole‐body and regional body fat and muscle mass with cardiovascular disease (CVD) and non‐CVD mortality in type 2 diabetes (T2D).

**Methods:**

Within the National Health and Nutrition Examination Survey 1999–2006, 1417 adults aged ≥50 years with T2D were selected. Dual‐energy X‐ray absorptiometry was used to derive whole‐body, trunk, arm, and leg fat mass and muscle mass indices (FMI and MMI). Mortality data until 31 December 2019 were retrieved from the National Death Index. Hazard ratios (HRs) and 95% confidence intervals (CIs) were estimated from Cox proportional hazard models.

**Results:**

A total of 1417 participants were included in this study (weighted mean age [standard error]: 63.7 [0.3] years; 50.5% female). Over a median follow‐up of 13.6 years, 797 deaths were recorded (371 CVD‐related and 426 non‐CVD deaths). Higher FMI in the arm was associated with increased risk of non‐CVD mortality (fourth quartile [Q4] vs. first quartile [Q1]: HR 1.82 [95% CI 1.13–2.94]), whereas higher FMI in the trunk or leg was not significantly associated with CVD or non‐CVD mortality. Conversely, higher arm MMI was associated with a lower risk of both CVD (Q4 vs. Q1: HR 0.51 [95% CI 0.33–0.81]) and non‐CVD (Q4 vs. Q1: HR 0.56 [95% CI 0.33–0.94]) mortality. There was a significant interaction between smoking status and arm FMI on non‐CVD mortality (*P* for interaction = 0.007). Higher arm FMI was associated with a higher risk of non‐CVD mortality among current or former smokers (Q4 vs. Q1: HR 2.67 [95% CI 1.46–4.88]) but not non‐smokers (Q4 vs. Q1: HR 0.85 [95% CI 0.49–1.47]).

**Conclusions:**

Fat mass and muscle mass, especially in the arm, are differently associated with CVD and non‐CVD mortality in people with T2D. Our findings underscore the predictive value of body compositions in the arm in forecasting mortality among older adults with T2D.

## Introduction

The current prevalence of type 2 diabetes (hereafter referred to as diabetes) in the United States stands at 38.4 million individuals, constituting 11.6% of the population in 2021, and is expected to escalate rapidly.^1^ Diabetes is the eighth leading cause of death in the United States, resulting in over 100 000 deaths in 2021 alone.^1^ Besides cardiovascular disease (CVD)‐related (e.g., coronary artery disease, heart failure and stroke) mortality,^2^ diabetes has also been linked to non‐CVD mortality such as chronic kidney disease and cancer.^2^, ^3^ Diabetes commonly coexists with overweight or obesity (defined as a body mass index [BMI] of 25 kg/m^2^ or higher) in 89.8% of cases.^1^ Excessive fat accumulation, seen in overweight or obesity, has been linked to increased mortality in the general population.^4^ Interestingly, and opposite to what is expected, among individuals with diabetes, comorbid overweight/obesity can exhibit a survival advantage over those with normal weight—a phenomenon known as the ‘obesity paradox’.^5^, ^6^, ^7^

This paradox may stem from imprecise adiposity measures, mainly the reliance on proxy measures, with most of the studies using BMI to define obesity,^5^, ^7^ rather than objective and more precise measurements of fat mass such as those derived from dual‐energy X‐ray absorptiometry (DXA) metrics. A drawback of BMI is its incorporation of not only fat mass but also muscle mass and its incapability of discerning between these masses that correlate differently with the ageing process. Research indicates that high fat mass positively correlates with mortality, while high muscle mass shows the inverse, that is, decreased mortality.^8^ This becomes crucial for older populations, where muscle mass tends to decrease with age while fat mass increases,^9^ leading to higher fat deposits in visceral compartments, elevating the risk of health‐related comorbidities and mortality.^10^ Consequently, a normal late‐life BMI (20–24.9 kg/m^2^) consisting of high fat mass but low muscle mass may exhibit a negative impact on health, while a higher BMI, indicating a high level of muscle mass, may indicate a positive correlation with health, such as in the obesity paradox. Moreover, BMI fails to consider the local distributions of fat mass and muscle mass, overlooking variations in biological functions across different body locations. For instance, altered glucose metabolism and insulin resistance, core features of diabetes, have shown associations with body composition in different regions. A study found that high trunk fat mass was linked with altered glucose metabolism, whereas high fat mass in the legs was associated with a decreased risk of insulin resistance.^11^ Moreover, an experimental study revealed a higher glucose clearance in the arm than in the leg muscle, indicating variations in regional tissue metabolism and muscle properties.^12^

Despite growing evidence supporting associations between body compositions (e.g., fat and muscle mass) and their regional distributions with glucose metabolism,^11^, ^12^ the understanding of how whole‐body and regional body fat and muscle composition is related to all‐cause and cause‐specific mortality in diabetes remains limited. To address these gaps, we investigated the associations of whole‐body and regional body fat and muscle mass with all‐cause, CVD and non‐CVD mortality among older adults with diabetes from the National Health and Nutrition Examination Survey (NHANES).

## Methods

### Study population

The NHANES is a national survey with a complex and multi‐stage sampling design to randomly select a nationally representative population from noninstitutionalized US residents.^13^ In the current study, data from four survey cycles (1999–2000, 2001–2002, 2003–2004 and 2005–2006) were used (*N* = 41 474). Diabetes status was ascertained at each survey cycle based on self‐reported diagnosis, fasting blood glucose ≥126 mg/dL, glycated haemoglobin (HbA1c) ≥6.5%, or use of insulin or oral hypoglycaemic medications.^14^ Among 2693 participants with diabetes, we further excluded participants diagnosed with diabetes before age 25 (indicating plausible type 1 diabetes, *n* = 210), pregnant or lactating (*n* = 14), aged <50 years at the study entry (*n* = 432), without DXA‐scanned body compositions (*n* = 392) or without eligible death status (*n* = 228). Finally, 1417 participants aged ≥50 years with diabetes and with DXA‐scanned body compositions were included in the analyses.

The Institutional Review Board for the National Center for Health Statistics approved the NHANES, which was performed in accordance with the Declaration of Helsinki. All participants provided written informed consent before data collection.

### Data collection and assessment of covariates

Information on socio‐demographic factors (i.e., age, sex, race/ethnicity and education), smoking status, physical activity and medical history was collected using standard questionnaires during the household interview.

Weight and height were measured by trained staff in the mobile examination centre. BMI was calculated using weight (kg) divided by squared height (m^2^) and was categorized into four groups, including underweight (<20.0 kg/m^2^), normal (20–24.9 kg/m^2^), overweight (25.0–29.9 kg/m^2^) and obese (≥30.0 kg/m^2^). Race/ethnicity includes non‐Hispanic white, non‐Hispanic black, Mexican American and other (e.g., other Hispanic and multi‐racial). Education was categorized based on the highest degree received: less than high school, high school or equivalent, or college or above. Smoking status was categorized as current, former or never smoker. Physical activity was categorized as active (≥150 min/week for moderate‐intensity activity or ≥75 min/week for vigorous‐intensity activity) or inactive (detailed information shown in *Text* *S1*). Hypertension was defined based on self‐reported medical history, the use of antihypertensive treatment, or a systolic (SBP) and diastolic blood pressure (DBP) ≥140/90 mmHg. At baseline, CVDs included history of any congestive heart failure, coronary heart disease, angina pectoris, heart attack or stroke. Diabetes treatment, including the use of insulin or oral hypoglycaemic medications, was self‐reported by participants. Controlled diabetes was defined as HbA1c levels <6.5% among people with diabetes. The duration of diabetes was calculated as the difference between the age of diabetes onset and the baseline age, categorized as <8 versus ≥8 years.

### Assessment of body composition by dual‐energy X‐ray absorptiometry

Whole‐body DXA scans were conducted using a Hologic QDR 4500A fan‐beam densitometer (Hologic, Inc., Bedford, MA) to measure whole‐body and regional body (i.e., trunk, arm and leg) fat and muscle mass.^15^ Fat mass index (FMI) was calculated as whole‐body or regional body fat mass (kg) divided by height squared (m^2^). Muscle mass index (MMI) was calculated as whole‐body or regional body muscle mass (kg) divided by height squared (m^2^). Detailed calculations are described in *Text* *S2*. Participants were grouped into quartiles according to sex‐specified values of FMI and MMI at the 25th, 50th and 75th percentile. To create four combinations of fat and muscle mass (high fat mass and low muscle mass, low fat mass and low muscle mass, high fat mass and high muscle mass, and low fat mass and high muscle mass), fat and muscle mass were dichotomized using the median value to create lower and higher groups.

### Assessment of mortality

Mortality information up to 31 December 2019 was ascertained from the National Death Index (NDI). Data about the underlying cause of death were coded using the International Classification of Diseases, Tenth Revision. CVD mortality included heart diseases (Codes I00–I09, I11, I13 and I20–I51), cerebrovascular diseases (I60–I69) and diabetes‐related death (E10–E14). Non‐CVD mortality was defined as all other deaths, including cancer (C00–C97), chronic lower respiratory diseases (J40–J47), accidents/unintentional injuries (V01–X59, Y85 and Y86), Alzheimer's disease (G30), influenza and pneumonia (J09–J18), nephritis, nephrotic syndrome and nephrosis (N00–N07, N17–N19 and N25–N27), and all other causes. The proportion of cause‐specific deaths has been illustrated in the pie chart (*Figure* *S1*).

### Statistical analysis

To account for the NHANES survey design, the 8‐year sampling weights of four survey cycles were calculated appropriately^16^ and incorporated into all analyses.

Cox proportional hazard models were used to estimate hazard ratios (HRs) and 95% confidence intervals (CIs) for the associations of body compositions in quartiles with all‐cause, CVD and non‐CVD mortality. Schoenfeld residuals were calculated to test the proportional hazard assumption, resulting in no violations. Follow‐up time was used as the timescale and calculated as the time from the initial date of the NHANES survey cycle until the date of death or 31 December 2019, whichever occurred first. In the Cox models, we adjusted for age, sex, race/ethnicity and education (Model 1) and subsequently adjusted for smoking status, physical activity, diabetes treatment, hypertension and CVD (Model 2). Moreover, the FMI and MMI of the corresponding regions were mutually adjusted. For example, we adjusted for arm FMI when exploring the association between arm MMI and mortality. We further examined the associations of the combinations of fat and muscle mass with all‐cause, CVD and non‐CVD mortality. We explored the interaction between age, sex, smoking status, physical activity, diabetes control status and diabetes duration with body compositions by adding their cross‐product terms into the model to test effect modification. We further conducted stratified analyses by baseline age groups (<65 vs. ≥65 years), sex (male vs. female), smoking status (current or former smoker vs. non‐smoker), physical activity (inactive vs. active), diabetes control status (controlled vs. uncontrolled diabetes) and diabetes duration (<8 vs. ≥8 years) to explore the associations between regional body compositions (only including extreme quartiles, highest [fourth quartile—Q4] vs. lowest [first quartile—Q1]) and mortality.

In the supplementary analysis, we explored the associations between baseline BMI categories and all‐cause, CVD and non‐CVD mortality. We conducted Kaplan–Meier survival analyses to visually illustrate the probability of mortality occurring over time across different groups of body compositions. All statistical analyses were performed using SAS software (Version 9.4, SAS Institute, Cary, NC) with the SURVEYFREQ, SURVEYMEANS or SURVEYPHREG procedure. All *P* values were two‐sided, and statistical significance was defined as *P* < 0.05. Missing or invalid DXA data were imputed five times in NHANES, resulting in five DXA datasets. The imputations were performed using IVEware based on the sequential regression imputation method.^17^, ^18^ Analyses were performed within each imputed DXA dataset, and then five estimates were pooled to derive a single composite estimate.

## Results

There were 1417 participants aged ≥50 years with diabetes included in this study (weighted mean age [standard error—SE]: 63.7 [0.3] years; 50.5% female). During a median follow‐up of 13.6 (inter‐quartile range: 7.6–16.3) years, 797 deaths were documented, including 371 CVD‐related deaths and 426 non‐CVD deaths.

Compared to participants who died during the follow‐up, those alive were more likely to be younger, female, never smokers, with a higher level of education and physical activity, and less likely to be non‐Hispanic white and to have hypertension or CVD (*P* < 0.05 for all) (*Table* *S1*). Compared to participants who died from CVD, those who died from non‐CVD causes were more likely to be current or former smokers and less likely to have diabetes treatment or prevalent hypertension (*P* < 0.05 for all) (*Table* *S2*).

### Associations of whole‐body and regional body fat mass index with all‐cause, cardiovascular disease and non‐cardiovascular disease mortality

The HR (95% CI) of all‐cause mortality comparing the Q4 to the Q1 was 1.51 (1.03–2.22) for whole‐body FMI, 1.47 (1.04–2.09) for appendicular FMI and 1.66 (1.16–2.37) for arm FMI after additionally adjusting for whole‐body or corresponding regional body MMI and other confounders including age, sex, race/ethnicity, education, smoking status, physical activity, anti‐diabetic medications, hypertension and CVD (i.e., Model 2) (*Table* *1*). Both trunk and leg FMI were not significantly associated with all‐cause mortality (*P* for all >0.05). However, compared to the lowest quartile, the highest quartiles for neither whole‐body nor regional body FMI were significantly associated with CVD mortality. The HR (95% CI) of non‐CVD mortality comparing the Q4 to the Q1 was 1.65 (1.02–2.67) for appendicular FMI and 1.82 (1.13–2.94) for arm FMI.

**Table 1:** HR (95% CI) for all‐cause, CVD and non‐CVD mortality according to quartiles of whole‐body and regional body FMI among participants with type 2 diabetes

Body composition	Quartile for body composition, HR (95% CI)			
	Q1	Q2	Q3	Q4
For all‐cause mortality				
Whole‐body FMI				
Range, female	≤10.9	>10.9, ≤13.3	>13.3, ≤16.7	>16.7
Range, male	≤7.5	>7.5, ≤9.3	>9.3, ≤11.4	>11.4
Model 1	Reference	1.16 (0.89–1.49)	1.09 (0.85–1.39)	**1.33 (1.01–1.75)**
Model 2	Reference	1.29 (0.93–1.79)	1.23 (0.93–1.64)	**1.51 (1.03–2.22)**
Trunk FMI				
Range, female	≤5.6	>5.6, ≤6.9	>6.9, ≤8.7	>8.7
Range, male	≤4.1	>4.1, ≤5.2	>5.2, ≤6.4	>6.4
Model 1	Reference	0.92 (0.71–1.20)	1.03 (0.80–1.32)	1.30 (0.99–1.70)
Model 2	Reference	1.06 (0.77–1.46)	1.14 (0.86–1.51)	1.34 (0.89–2.04)
Appendicular FMI				
Range, female	≤4.7	>4.7, ≤6.0	>6.0, ≤7.6	>7.6
Range, male	≤2.9	>2.9, ≤3.6	>3.6, ≤4.6	>4.6
Model 1	Reference	1.11 (0.84–1.45)	1.16 (0.89–1.50)	1.22 (0.93–1.61)
Model 2	Reference	1.23 (0.92–1.64)	**1.35 (1.02–1.78)**	**1.47 (1.04–2.09)**
Leg FMI				
Range, female	≤3.2	>3.2, ≤4.2	>4.2, ≤5.4	>5.4
Range, male	≤2.0	>2.0, ≤2.5	>2.5, ≤3.2	>3.2
Model 1	Reference	1.11 (0.83–1.48)	1.15 (0.89–1.47)	1.21 (0.90–1.63)
Model 2	Reference	1.18 (0.86–1.60)	1.27 (0.95–1.69)	1.27 (0.89–1.83)
Arm FMI				
Range, female	≤1.4	>1.4, ≤1.8	>1.8, ≤2.2	>2.2
Range, male	≤0.9	>0.9, ≤1.1	>1.1, ≤1.4	>1.4
Model 1	Reference	1.08 (0.83–1.40)	1.19 (0.97–1.47)	1.29 (0.99–1.68)
Model 2	Reference	1.23 (0.96–1.57)	**1.42 (1.13–1.78)**	**1.66 (1.16–2.37)**
For CVD mortality				
Whole‐body FMI				
Model 1	Reference	1.40 (0.99–1.98)	0.99 (0.67–1.46)	1.38 (0.93–2.04)
Model 2	Reference	**1.50 (1.00–2.27)**	1.07 (0.70–1.65)	1.43 (0.87–2.37)
Trunk FMI				
Model 1	Reference	1.15 (0.82–1.62)	1.02 (0.72–1.45)	1.35 (0.90–2.02)
Model 2		1.27 (0.83–1.96)	1.06 (0.70–1.60)	1.33 (0.73–2.39)
Appendicular FMI				
Model 1	Reference	1.07 (0.73–1.55)	1.05 (0.72–1.53)	1.14 (0.77–1.69)
Model 2	Reference	1.15 (0.77–1.72)	1.21 (0.81–1.81)	1.27 (0.80–2.04)
Leg FMI				
Model 1	Reference	1.11 (0.73–1.69)	1.02 (0.72–1.45)	1.10 (0.71–1.68)
Model 2	Reference	1.14 (0.72–1.83)	1.08 (0.73–1.59)	1.05 (0.64–1.71)
Arm FMI				
Model 1	Reference	1.08 (0.79–1.46)	1.14 (0.82–1.59)	1.25 (0.87–1.79)
Model 2	Reference	1.29 (0.94–1.76)	1.32 (0.92–1.90)	1.47 (0.93–2.33)
For non‐CVD mortality				
Whole‐body FMI				
Model 1	Reference	0.94 (0.63–1.40)	1.17 (0.83–1.66)	1.29 (0.91–1.83)
Model 2	Reference	1.09 (0.68–1.74)	1.36 (0.93–1.98)	1.57 (0.98–2.53)
Trunk FMI				
Model 1	Reference	0.73 (0.50–1.08)	1.03 (0.72–1.46)	1.25 (0.89–1.76)
Model 2		0.88 (0.58–1.34)	1.20 (0.81–1.79)	1.36 (0.82–2.24)
Appendicular FMI				
Model 1	Reference	1.15 (0.77–1.71)	1.26 (0.87–1.83)	1.31 (0.91–1.87)
Model 2	Reference	1.28 (0.80–2.04)	1.46 (0.96–2.22)	**1.65 (1.02–2.67)**
Leg FMI				
Model 1	Reference	1.11 (0.73–1.68)	1.28 (0.85–1.91)	1.33 (0.90–1.97)
Model 2	Reference	1.18 (0.76–1.84)	1.45 (0.94–2.25)	1.48 (0.90–2.44)
Arm FMI				
Model 1	Reference	1.09 (0.73–1.63)	1.25 (0.88–1.77)	1.33 (0.94–1.88)
Model 2	Reference	1.17 (0.80–1.71)	**1.52 (1.05–2.19)**	**1.82 (1.13–2.94)**

*Note*: Model 1 was adjusted for age (continuous), sex (female or male), race/ethnicity (non‐Hispanic white, non‐Hispanic black, Mexican American or other) and education (less than high school, high school or equivalent, or college or above). Model 2 was further adjusted for smoking status (never smoker, former smoker or current smoker), physical activity (inactive or active), anti‐diabetic medications (no or yes), hypertension (no or yes), cardiovascular disease (no or yes) and corresponding muscle mass index. Statistically significant results are highlighted in bold font in the table. Abbreviations: CI, confidence interval; CVD, cardiovascular disease; FMI, fat mass index; HR, hazard ratio; Q1, first quartile; Q2, second quartile; Q3, third quartile; Q4, fourth quartile.

### Associations of whole‐body and regional body muscle mass index with all‐cause, cardiovascular disease and non‐cardiovascular disease mortality

Compared with the Q1, the second quartile (Q2) and the third quartile (Q3) of whole‐body MMI showed a reduced all‐cause mortality risk after adjusting for FMI and other confounders in Model 2 (HR 0.74 [95% CI 0.56–0.97] and HR 0.72 [95% CI 0.52–0.99], respectively), and the Q4 tended to be associated with decreased risk of all‐cause mortality (HR 0.74 [95% CI 0.52–1.05], *P* = 0.091), although not statistically significant (*Table* *2*). When examining specific regions, compared to the Q1, the higher quartiles of appendicular MMI (Q2–Q4), leg MMI (both Q2 and Q3) and arm MMI (Q3 and Q4) were associated with reduced all‐cause mortality risk. However, trunk MMI showed a non‐significant association with mortality. For CVD mortality, the higher quartiles of whole‐body MMI (Q2), appendicular MMI (Q3) and arm MMI (Q4) were associated with decreased CVD mortality. For non‐CVD mortality, the Q4 of arm MMI was associated with decreased mortality risk (Q4 vs. Q1: HR 0.56 [95% CI 0.33–0.94]) but no other regional body MMI.

**Table 2:** HR (95% CI) for all‐cause, CVD and non‐CVD mortality according to quartiles of whole‐body and regional body MMI among participants with type 2 diabetes

Body composition	Quartile for body composition, HR (95% CI)			
	Q1	Q2	Q3	Q4
For all‐cause mortality				
Whole‐body MMI				
Range, female	≤15.3	>15.3, ≤17.0	>17.0, ≤18.8	>18.8
Range, male	≤17.8	>17.8, ≤19.5	>19.5, ≤21.4	>21.4
Model 1	Reference	0.85 (0.65–1.11)	0.83 (0.63–1.09)	1.05 (0.80–1.38)
Model 2	Reference	**0.74 (0.56–0.97)**	**0.72 (0.52–0.99)**	0.74 (0.52–1.05)
Trunk MMI				
Range, female	≤8.1	>8.1, ≤8.8	>8.8, ≤9.8	>9.8
Range, male	≤9.1	>9.1, ≤9.9	>9.9, ≤10.9	>10.9
Model 1	Reference	0.82 (0.65–1.04)	0.85 (0.64–1.12)	1.17 (0.85–1.61)
Model 2	Reference	0.77 (0.59–1.02)	0.79 (0.58–1.08)	0.86 (0.55–1.35)
Appendicular MMI				
Range, female	≤6.1	>6.1, ≤6.9	>6.9, ≤7.9	>7.9
Range, male	≤7.5	>7.5, ≤8.3	>8.3, ≤9.3	>9.3
Model 1	Reference	**0.76 (0.60–0.97)**	**0.70 (0.53–0.94)**	0.83 (0.64–1.07)
Model 2	Reference	**0.69 (0.53–0.88)**	**0.66 (0.49–0.88)**	**0.66 (0.49–0.88)**
Leg MMI				
Range, female	≤4.5	>4.5, ≤5.1	>5.1, ≤5.9	>5.9
Range, male	≤5.3	>5.3, ≤5.9	>5.9, ≤6.6	>6.6
Model 1	Reference	**0.77 (0.61–0.97)**	**0.74 (0.56–0.99)**	0.94 (0.73–1.20)
Model 2	Reference	**0.68 (0.54–0.87)**	**0.71 (0.53–0.95)**	0.80 (0.60–1.07)
Arm MMI				
Range, female	≤1.5	>1.5, ≤1.7	>1.7, ≤2.0	>2.0
Range, male	≤2.1	>2.1, ≤2.4	>2.4, ≤2.7	>2.7
Model 1	Reference	0.93 (0.74–1.17)	**0.68 (0.51–0.91)**	**0.75 (0.58–0.97)**
Model 2	Reference	0.84 (0.64–1.10)	**0.62 (0.45–0.84)**	**0.54 (0.38–0.76)**
For CVD mortality				
Whole‐body MMI				
Model 1	Reference	0.85 (0.60–1.20)	0.99 (0.65–1.52)	1.10 (0.71–1.71)
Model 2	Reference	0.65 (0.43–1.00)	0.80 (0.49–1.31)	0.71 (0.42–1.22)
Trunk MMI				
Model 1	Reference	0.85 (0.57–1.28)	1.02 (0.65–1.60)	1.18 (0.74–1.89)
Model 2	Reference	0.71 (0.44–1.14)	0.91 (0.55–1.51)	0.80 (0.42–1.52)
Appendicular MMI				
Model 1	Reference	0.76 (0.47–1.20)	0.67 (0.44–1.02)	0.88 (0.56–1.37)
Model 2	Reference	0.65 (0.40–1.08)	**0.61 (0.39–0.94)**	0.67 (0.40–1.15)
Leg MMI				
Model 1	Reference	0.72 (0.46–1.14)	0.73 (0.49–1.08)	0.99 (0.64–1.52)
Model 2	Reference	0.85 (0.57–1.28)	1.02 (0.65–1.60)	1.18 (0.74–1.89)
Arm MMI				
Model 1	Reference	0.90 (0.59–1.36)	0.77 (0.49–1.21)	0.74 (0.51–1.08)
Model 2	Reference	0.75 (0.44–1.28)	0.67 (0.40–1.10)	**0.51 (0.33–0.81)**
For non‐CVD mortality				
Whole‐body MMI				
Model 1	Reference	0.84 (0.54–1.32)	0.69 (0.45–1.04)	1.01 (0.69–1.46)
Model 2	Reference	0.83 (0.54–1.29)	0.66 (0.41–1.06)	0.77 (0.46–1.29)
Trunk MMI				
Model 1	Reference	0.79 (0.57–1.09)	0.71 (0.47–1.06)	1.15 (0.76–1.75)
Model 2	Reference	0.83 (0.57–1.20)	0.69 (0.44–1.08)	0.91 (0.48–1.71)
Appendicular MMI				
Model 1	Reference	0.77 (0.56–1.06)	0.73 (0.47–1.16)	0.79 (0.57–1.10)
Model 2	Reference	0.72 (0.53–1.00)	0.73 (0.44–1.21)	0.65 (0.41–1.03)
Leg MMI				
Model 1	Reference	0.81 (0.57–1.16)	0.76 (0.51–1.13)	0.89 (0.64–1.25)
Model 2	Reference	0.73 (0.51–1.05)	0.73 (0.49–1.10)	0.76 (0.48–1.22)
Arm MMI				
Model 1	Reference	0.96 (0.68–1.36)	**0.61 (0.39–0.95)**	0.75 (0.54–1.06)
Model 2	Reference	0.91 (0.64–1.30)	0.57 (0.33–1.01)	**0.56 (0.33–0.94)**

*Note*: Model 1 was adjusted for age (continuous), sex (female or male), race/ethnicity (non‐Hispanic white, non‐Hispanic black, Mexican American or other) and education (less than high school, high school or equivalent, or college or above). Model 2 was further adjusted for smoking status (never smoker, former smoker or current smoker), physical activity (inactive or active), anti‐diabetic medications (no or yes), hypertension (no or yes), cardiovascular disease (no or yes) and corresponding fat mass index. Statistically significant results are highlighted in bold font in the table. Abbreviations: CI, confidence interval; CVD, cardiovascular disease; HR, hazard ratio; MMI, muscle mass index; Q1, first quartile; Q2, second quartile; Q3, third quartile; Q4, fourth quartile.

### Joint effect of fat mass index and muscle mass index on mortality

Compared to participants with high arm FMI and low arm MMI, those exhibiting low FMI and low MMI, high FMI and high MMI, and low FMI and high MMI had a lower risk of all‐cause mortality (*Table* *3*). Compared to participants with high whole‐body FMI and low whole‐body MMI, those with low FMI and high MMI had a lower risk of non‐CVD mortality (HR 0.54 [95% CI 0.29–0.99]). Compared to participants with high arm FMI and low arm MMI, those exhibiting low FMI and low MMI, high FMI and high MMI, and low FMI and high MMI were associated with decreased non‐CVD mortality. No statistically significant associations were observed between the combinations of FMI and MMI with CVD mortality.

**Table 3:** HR (95% CI) for all‐cause, CVD and non‐CVD mortality according to the combination of fat mass and muscle mass

Body composition	HR (95% CI)		
	For all‐cause mortality	For CVD mortality	For non‐CVD mortality
Whole body			
High fat mass and low muscle mass	Reference	Reference	Reference
Low fat mass and low muscle mass	1.11 (0.89–1.40)	1.51 (0.99–2.32)	0.90 (0.65–1.25)
High fat mass and high muscle mass	1.19 (0.91–1.56)	1.51 (0.89–2.55)	1.02 (0.77–1.36)
Low fat mass and high muscle mass	0.82 (0.57–1.20)	1.36 (0.80–2.30)	**0.54 (0.29–0.99)**
Trunk			
High fat mass and low muscle mass	Reference	Reference	Reference
Low fat mass and low muscle mass	0.96 (0.73–1.27)	1.04 (0.65–1.66)	0.90 (0.63–1.30)
High fat mass and high muscle mass	1.10 (0.83–1.46)	1.12 (0.66–1.91)	1.10 (0.81–1.49)
Low fat mass and high muscle mass	0.85 (0.58–1.26)	1.22 (0.70–2.11)	0.60 (0.34–1.04)
Leg			
High fat mass and low muscle mass	Reference	Reference	Reference
Low fat mass and low muscle mass	1.02 (0.74–1.39)	1.14 (0.67–1.96)	0.92 (0.64–1.31)
High fat mass and high muscle mass	1.08 (0.82–1.41)	1.08 (0.65–1.80)	1.07 (0.76–1.51)
Low fat mass and high muscle mass	0.82 (0.55–1.23)	1.02 (0.54–1.93)	0.66 (0.37–1.17)
Arm			
High fat mass and low muscle mass	Reference	Reference	Reference
Low fat mass and low muscle mass	**0.73 (0.58–0.93)**	0.87 (0.58–1.30)	**0.63 (0.46–0.87)**
High fat mass and high muscle mass	**0.66 (0.53–0.82)**	0.75 (0.50–1.11)	**0.59 (0.41–0.84)**
Low fat mass and high muscle mass	**0.53 (0.37–0.76)**	0.64 (0.40–1.04)	**0.46 (0.25–0.84)**

*Note*: Model was adjusted for age (continuous), sex (female or male), race/ethnicity (non‐Hispanic white, non‐Hispanic black, Mexican American or other), education (less than high school, high school or equivalent, or college or above), smoking status (never smoker, former smoker or current smoker), physical activity (inactive or active), anti‐diabetic medications (no or yes), hypertension (no or yes) and cardiovascular disease (no or yes). Statistically significant results are highlighted in bold font in the table. Abbreviations: CI, confidence interval; CVD, cardiovascular disease; HR, hazard ratio.

### The role of age, sex, lifestyles and diabetes features

**Figure 1:**
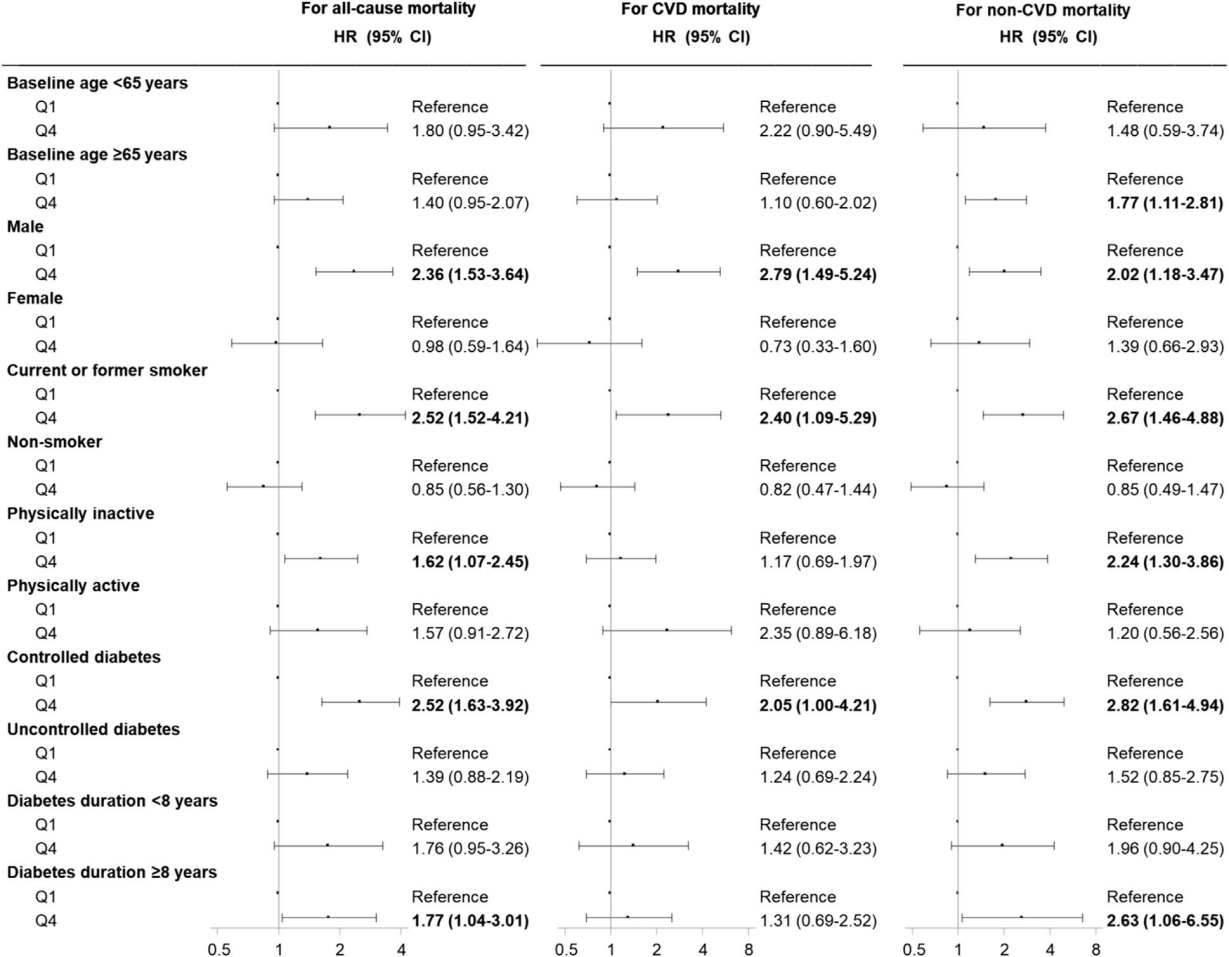
Hazard ratio (HR) (95% confidence interval [CI]) for all‐cause, cardiovascular disease (CVD) and non‐CVD mortality according to the extreme quartiles of arm fat mass index (FMI) across age, sex, smoking status, physical activity, diabetes control status and diabetes duration. Model was adjusted for age (continuous), sex (female or male), race/ethnicity (non‐Hispanic white, non‐Hispanic black, Mexican American or other), education (less than high school, high school or equivalent, or college or above), smoking status (never smoker, former smoker or current smoker), physical activity (inactive or active), anti‐diabetic medications (no or yes), hypertension (no or yes), cardiovascular disease (no or yes) and arm muscle mass index if applicable. We bolded results when *P* < 0.05. There were no significant multiplicative interactions (all *P* values >0.05) between age, sex, smoking status, physical activity, glycaemic control, diabetes duration and arm FMI on all‐cause, CVD and non‐CVD mortality, except that there were significant interactions between smoking status and arm FMI on all‐cause and non‐CVD mortality (*P* value for interaction = 0.020 and 0.007, respectively). Q1, first quartile; Q4, fourth quartile.

**Figure 2:**
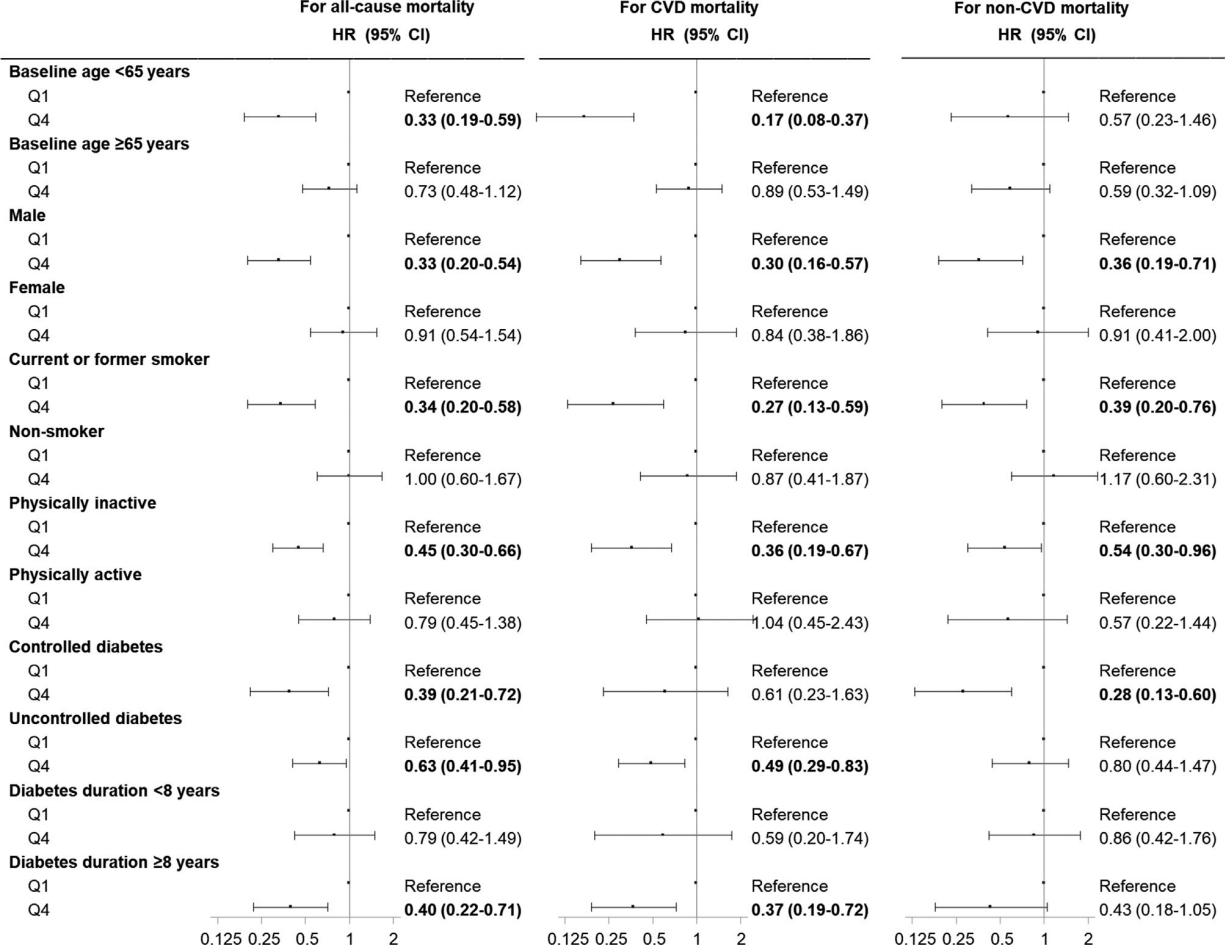
Hazard ratio (HR) (95% confidence interval [CI]) for all‐cause, cardiovascular disease (CVD) and non‐CVD mortality according to the extreme quartiles of arm muscle mass index (MMI) across age, sex, smoking status, physical activity, diabetes control status and diabetes duration. Model was adjusted for age (continuous), sex (female or male), race/ethnicity (non‐Hispanic white, non‐Hispanic black, Mexican American or other), education (less than high school, high school or equivalent, or college or above), smoking status (never smoker, former smoker or current smoker), physical activity (inactive or active), anti‐diabetic medications (no or yes), hypertension (no or yes), cardiovascular disease (no or yes) and arm fat mass index if applicable. We bolded results when *P* < 0.05. There were no significant multiplicative interactions (all *P* values >0.05) between age, sex, smoking status, physical activity, glycaemic control, diabetes duration and arm MMI on all‐cause, CVD and non‐CVD mortality. Q1, first quartile; Q4, fourth quartile.

There were no significant multiplicative interactions (all *P* values >0.05) between age, sex, smoking status, physical activity, diabetes features and arm body compositions on all‐cause, CVD and non‐CVD mortality, except that there were significant interactions between smoking status and arm FMI on all‐cause and non‐CVD mortality (*P* value for interaction = 0.020 and 0.007, respectively). We performed a series of stratified analyses across chronological age, biological sex, smoking status, physical activity, glycaemic control and diabetes duration to explore the associations of FMI (*Figure* *1*) and MMI (*Figure* *2*) with all‐cause, CVD and non‐CVD mortality.

The Q4 of arm FMI was significantly associated with all‐cause mortality in males, current or former smokers, those who were physically inactive, those with controlled diabetes and those with a longer duration of diabetes (*Figure* *1*). The Q4 of arm FMI was significantly associated with increased CVD mortality in males, current or former smokers, and those with controlled diabetes. The Q4 of arm FMI was significantly associated with increased non‐CVD mortality in older adults (age ≥65 years), males, current or former smokers, those who were physically inactive, those with controlled diabetes and those with a longer duration of diabetes.

The Q4 of arm MMI was significantly associated with decreased all‐cause mortality in younger adults (age <65 years), males, current or former smokers, those who were physically inactive, those with controlled or uncontrolled diabetes, and those with a longer duration of diabetes (*Figure* *2*). The Q4 of arm MMI was significantly associated with decreased CVD mortality in younger adults (age <65 years), males, current or former smokers, those who were physically inactive, those with uncontrolled diabetes and those with a longer duration of diabetes. The Q4 of arm MMI was significantly associated with decreased non‐CVD mortality in males, current or former smokers, those who were physically inactive and those with controlled diabetes.

### Supplementary results

Compared to the normal BMI group, being underweight was associated with an increased risk of all‐cause and non‐CVD mortality but not CVD mortality. Overweight and obesity were not significantly associated with all‐cause, CVD or non‐CVD mortality among older adults with diabetes (*Table* *S3*). *Figures* *S2*–*S4* present the Kaplan–Meier curves illustrating overall survival, survival free from CVD mortality and survival free from non‐CVD mortality across arm FMI and arm MMI groups.

## Discussion

This longitudinal study of older adults with diabetes in the United States highlighted the following main findings. First, higher whole‐body fat mass was associated with increased all‐cause mortality, whereas greater muscle mass was associated with reduced risk. Second, the associations of fat and muscle mass with mortality were contingent on their location. Higher fat mass in the arm, but not in the trunk or leg, was associated with an increased risk of all‐cause and non‐CVD mortality but not CVD mortality. Conversely, higher muscle mass in the arm was associated with a lowered risk of all‐cause, CVD and non‐CVD mortality. Third, compared to participants with high fat mass and low muscle mass in the arm, those with either low fat mass or high muscle mass had lower all‐cause and non‐CVD mortality. Fourth, the associations of arm FMI with all‐cause and non‐CVD mortality were statistically significant among males, current/former smokers, physically inactive individuals, those with controlled diabetes and those with a longer diabetes duration (≥8 years). The associations of arm MMI with all‐cause, CVD and non‐CVD mortality were statistically significant among males, current/former smokers and physically inactive individuals. Our findings underscore the predictive value of arm muscle mass, as opposed to fat mass or other regional body compositions, in indicating subsequent CVD and non‐CVD mortality among older adults with diabetes.

Previous studies addressed the relationship between body fat mass—directly measured by DXA or computed tomography (CT)—and mortality mainly in general populations.^19^, ^20^ So far, evidence on the link between body composition and mortality, especially cause‐specific mortality, among older adults with diabetes has been scarce and inconclusive. A study from the UK Biobank, including 23 842 persons, but mainly middle‐aged (aged 40–69 years), with diabetes, observed a U‐shaped association between bioelectrical impedance derived percentage of fat mass and all‐cause mortality, but only in males not in females.^5^ Conversely, a study conducted among 163 Japanese men (mean age 64.4 years) and 141 postmenopausal women (mean age 66.1 years) with diabetes showed no association between DXA‐derived fat mass percentage and mortality, in neither women nor men.^21^ Inconsistencies in these findings could be related to methodological issues such as the limited statistical power due to small sample sizes, possible residual confounding (due to unadjusted vascular risk factors, such as smoking status) or inappropriately assuming a linear association. Furthermore, a heightened percentage of fat mass may result from diminished muscle mass—a common occurrence in ageing and diabetes—rather than an actual increase in fat mass.^22^ To overcome this limitation, in the current study, we used DXA‐derived FMI instead of fat mass percentage to define obesity. Our study took a step further by delving into the associations between fat mass and CVD mortality and non‐CVD mortality among older adults with diabetes.

We found that a higher whole‐body FMI was associated with increased all‐cause mortality. We also observed that a higher FMI in the arm, but not in the trunk or leg, was associated with increased all‐cause and non‐CVD mortality. This indicated that fat mass in the arms may be a more robust anthropometric measure for predicting all‐cause and non‐CVD mortality among adults with diabetes. Moreover, these associations remained significant among males, current/former smokers, physically inactive participants and those with diabetes duration ≥8 years. We also observed that higher fat mass tended to be associated with increased mortality among physically active participants and those with shorter diabetes duration, albeit not significant. However, given the wide CI due to the limited sample size in subgroups, these results should be interpreted with caution. Future studies are warranted to explore which population may benefit more from managing fat mass.

Differences in mortality risk linked to fat distribution in different body locations are biologically plausible. Indeed, the adipose tissue of the upper body secretes more inflammatory cytokines than the adipose tissue in the lower body.^23^ Also, the upper‐body adipose tissue is more likely to release, but less likely to take up, free fatty acids during hyperinsulinaemia, further impairing the function of insulin.^24^, ^25^, ^26^ Arm fat mass seems to be more strongly associated with insulin resistance than the fat mass in the trunk or legs.^27^ This may also partly explain the better prediction of mortality observed in relation to fat mass in the arms, compared to other regions' fat mass in the current study sample. Nevertheless, more research is needed to better understand the mechanisms that underlie the different risks between the arm and other regional fat localizations.

Despite the reciprocal relationship between muscle mass and diabetes—in which low muscle mass leads to abnormal glucose metabolism, and reduced insulin signalling induces decreased muscle mass through reduced protein synthesis,^22^, ^28^ few studies have explored the associations between muscle mass and all‐cause mortality in older adults with diabetes.^21^, ^29^, ^30^ A prior study of Japanese adults with diabetes found that low muscle mass (defined as appendicular MMI < 7.0 kg/m^2^ for males and <5.4 kg/m^2^ for females) was associated with increased all‐cause mortality.^21^ In two other studies, greater psoas and paraspinous muscle mass measured by CT scans were also negatively associated with all‐cause mortality risk in African Americans^30^ and European Americans^29^ with diabetes. In line with previous studies, we found that higher appendicular muscle mass was associated with a reduced all‐cause mortality risk. Among the body's extremities, arm muscle mass had a stronger association with all‐cause mortality than leg muscle mass. Moreover, muscle mass in the arm but not in the trunk or leg was associated with a decreased risk of both CVD and non‐CVD mortality, and these associations persisted among males, current or former smokers, and those who were physically inactive. These findings underscore the importance of increasing arm muscle mass as an ideal target for strategies aiming to improve the prognosis of diabetes, in turn helping to avoid premature death, in older adults, especially if males have a history of unhealthy lifestyle habits. Further studies, possibly in an intervention setting, are warranted to confirm this hypothesis.

Reduced muscle mass has been associated with arterial stiffness and a decreased serum insulin‐like growth factor‐1 level, which can lead to increased mortality in diabetes.^31^, ^32^, ^33^ The different clinical impacts of arm and leg muscle mass on the prognosis of diabetes may be due to the higher glucose clearance regardless of insulin resistance and better preserved insulin sensitivity in the arm than leg muscle.^12^ Moreover, the heterogeneity effect in glucose clearance and insulin resistance of arm and leg muscle mass may be explained by their different muscle fibres—a higher proportion of fast fibres in the arm while a higher proportion of slow fibres in the leg. In diabetes, glucose transporter type 4 (GLUT4) is significantly higher in fast fibres compared with slow fibres.^34^ Future studies are warranted to further explore the role of arm muscle mass in the prognosis of diabetes.

### Strengths and limitations

The novelty of our study lies in the investigation of all‐cause, CVD and non‐CVD mortality risk in relation to whole‐body and regional body tissue composition, measured directly with DXA, in diabetes, based on a representative large study sample. Some limitations need to be acknowledged. First, although we included a wide range of covariates, there may be residual confounding due to unavailable factors, such as the severity of diabetes. Second, some variables, such as physical activity, were self‐reported, which may lead to misclassification, as physical activity is often over‐reported. However, a previous study using data from NHANES 2005–2006 validated the self‐reported physical activity questionnaire using device‐measured physical activity.^35^ Third, the associations between body compositions and mortality may vary by ethnicities. However, we could not conduct stratified analyses by ethnicity due to the limited sample size. Lastly, the present study was conducted among adults with diabetes, and the findings should be cautiously generalized to populations with other diseases.

In conclusion, our study in older adults with diabetes revealed that heightened whole‐body fat mass was associated with increased all‐cause mortality, whereas greater muscle mass was associated with a decreased risk. Notably, the associations between these body compositions and mortality exhibited location‐specific variations, with elevated arm fat mass, but not trunk or leg fat mass, linked to increased risks of all‐cause and non‐CVD mortality. Conversely, increased arm muscle mass was associated with decreased risks of all‐cause, CVD and non‐CVD mortality. Future studies are warranted to delve into the specific role of arm body compositions, particularly arm muscle mass, in predicting subsequent CVD and non‐CVD mortality among older adults with diabetes. This is crucial, as interventions targeting arm fat or muscle mass may hold potential benefits for the prognosis of diabetes.

## Conflict of interest statement

The authors declare no conflicts of interest.

## Funding

JG was supported by Lindhés Advokatbyrå AB (LA2023‐0065). YW was supported by the China Scholarship Council (201907930017). AM was supported by the Strategic Research Area Neuroscience (StratNeuro) at Karolinska Institutet, the Center for Innovative Medicine (Centrum för innovativ medicin [CIMED]) (FoUI‐988254), the Gamla Tjännarinor Foundation, the Loo och Hans Ostermans Foundation (2023‐01645) and the Foundation for Geriatric Diseases at Karolinska Institutet (2023‐01598).

## Supporting information


**Table S1.** Baseline characteristics of study population by mortality status at end of study (*N* = 1417).
**Table S2.** Baseline characteristics of study population with CVD or non‐CVD mortality at end of study (*N* = 797).
**Table S3.** HR (95% CI) for all‐cause, CVD, and non‐CVD mortality according to baseline BMI groups.
**Figure S1.** Proportion of cause‐specific deaths among participants aged ≥50 years with type 2 diabetes.
**Figure S2.** Kaplan–Meier curves showing overall survival across arm fat mass index and arm muscle mass index.
**Figure S3.** Kaplan–Meier curves showing survival from CVD mortality across arm fat mass index and arm muscle mass index.
**Figure S4.** Kaplan–Meier curves showing survival from non‐CVD mortality across arm fat mass index and arm muscle mass index.
**Text S1.** Data collection for physical activity.
**Text S2.** Calculations of body compositions.
