# Supplementary material for: Differential impacts of fat and muscle mass on cardiovascular and non‐cardiovascular mortality in individuals with type 2 diabetes
Source: J Cachexia Sarcopenia Muscle. 2024 Jul 12;15(5):1930–41. doi: 10.1002/jcsm.13542 (PMC11446681; doi:10.1002/jcsm.13542)
Supplement: Supplementary file 1 — Table S1. Baseline characteristics of study population by mortality status at end of study (N = 1417). Table S2. Baseline characteristics of study population with CVD or non‐CVD mortality at end of study (N = 797). Table S3. HR (95% CI) for all‐cause, CVD, and non‐CVD mortality according to baseline BMI groups. Figure S1. Proportion of cause‐specific deaths among participants aged ≥50 years with type 2 diabetes. Figure S2. Kaplan–Meier curves showing overall survival across arm fat mass index and arm muscle mass index. Figure S3. Kaplan–Meier curves showing survival from CVD mortality across arm fat mass index and arm muscle mass index. Figure S4. Kaplan–Meier curves showing survival from non‐CVD mortality across arm fat mass index and arm muscle mass index. Text S1. Data collection for physical activity. Text S2. Calculations of body compositions. [file JCSM-15-1930-s001.docx]

**Supplementary file**

**Table S1** Baseline characteristics of study population by mortality status at end of study (N = 1417)

**Table S2** Baseline characteristics of study population with CVD or non-CVD mortality at end of study (N = 797)

**Table S3** HR (95% CI) for all-cause, CVD, and non-CVD mortality according to baseline BMI groups

**Figure S1** Proportion of cause-specific deaths among participants aged ≥50 years with type 2 diabetes

**Figure S2** Kaplan–Meier curves showing overall survival across arm fat mass index and arm muscle mass index

**Figure S3** Kaplan–Meier curves showing survival from CVD mortality across arm fat mass index and arm muscle mass index

**Figure S4** Kaplan–Meier curves showing survival from non-CVD mortality across arm fat mass index and arm muscle mass index

**Text S1** Data collection for physical activity

**Text S2** Calculations of body compositions

**Table S1** Baseline characteristics of study population by mortality status at end of study (N = 1417)

| Characteristics | Total | Alive (N = 620) | Died (N = 797) | *P* value |
| --- | --- | --- | --- | --- |
| Age, years | 63.7 (0.3) | 59.7 (0.3) | 67.7 (0.5) | <0.001 |
| Sex (%) |  |  |  | 0.044 |
| Female | 50.5 | 53.6 | 47.5 |  |
| Male | 49.5 | 46.4 | 52.5 |  |
| Race/Ethnicity (%) |  |  |  | 0.002 |
| Mexican American | 6.6 | 8.0 | 5.2 |  |
| Non-Hispanic white | 67.2 | 62.3 | 72.2 |  |
| Non-Hispanic black | 15. 4 | 16.0 | 14.7 |  |
| Other | 10.8 | 13.7 | 7.9 |  |
| Highest education (%) |  |  |  | <0.001 |
| Less than high school | 31.7 | 24.4 | 39.0 |  |
| High school or equivalent | 25.5 | 26.5 | 24.5 |  |
| College or above | 42.8 | 49.1 | 36.5 |  |
| Smoking status (%) |  |  |  | <0.001 |
| Current | 16.2 | 14.3 | 18.1 |  |
| Former | 37.3 | 31.8 | 42.8 |  |
| Never | 46.5 | 53.9 | 39.1 |  |
| Physically active (%) |  |  |  | 0.005 |
| No | 67.9 | 62.9 | 73.0 |  |
| Yes | 32.1 | 37.1 | 27.0 |  |
| Diabetes treatment (oral or insulin) | 64.5 | 61.8 | 67.2 | 0.079 |
| Hypertension (%) | 72.5 | 68.8 | 76.2 | 0.014 |
| Cardiovascular disease (%) | 30.7 | 16.6 | 45.0 | <0.001 |

Data are presented as weighted mean (standard error) or weighted percentages.

**Table S2** Baseline characteristics of study population with CVD or non-CVD mortality at end of study (N = 797)

| Characteristics | CVD mortality (N = 371) | Non-CVD mortality (N = 426) | *P-value* |
| --- | --- | --- | --- |
| Age, years | 68.2 (0.76) | 67.2 (0.62) | 0.355 |
| Sex (%) |  |  | 0.268 |
| Female | 50.2 | 44.9 |  |
| Male | 49.8 | 55.1 |  |
| Race/Ethnicity (%) |  |  | 0.374 |
| Mexican American | 4.7 | 5.7 |  |
| Non-Hispanic white | 71.4 | 73.0 |  |
| Non-Hispanic black | 14.3 | 15.1 |  |
| Other | 9.6 | 6.2 |  |
| Highest education (%) |  |  | 0.221 |
| Less than high school | 42.7 | 35.5 |  |
| High school or equivalent | 22.5 | 26.4 |  |
| College or above | 34.8 | 38.1 |  |
| Smoking status (%) |  |  | <0.001 |
| Current | 13.7 | 22.2 |  |
| Former | 39.5 | 46.0 |  |
| Never | 46.8 | 31.8 |  |
| Physically active (%) |  |  | 0.100 |
| No | 69.3 | 76.6 |  |
| Yes | 30.7 | 23.4 |  |
| Diabetes treatment (oral or insulin) (%) | 74.5 | 60.3 | 0.003 |
| Hypertension (%) | 81.2 | 71.5 | 0.014 |
| Cardiovascular disease (%) | 49.7 | 40.6 | 0.068 |

Data are presented as weighted mean (standard error) or weighted percentages.

**Table S3** HR (95% CI) for all-cause, CVD, and non-CVD mortality according to baseline BMI groups

| **BMI group** | **HR (95% CI)** | | | | | |
| --- | --- | --- | --- | --- | --- | --- |
|  | **For all-cause mortality** | | **For CVD mortality** | | **For non-CVD mortality** | |
| Underweight | **3.15 (1.49-6.66)** | **0.003** | 1.30 (0.63-2.70) | 0.475 | **5.27 (1.93-14.4)** | **0.002** |
| Normal | Reference |  | Reference |  | Reference |  |
| Overweight | 0.99 (0.75-1.31) | 0.950 | 0.89 (0.60-1.33) | 0.561 | 1.12 (0.68-1.82) | 0.659 |
| Obese | 1.13 (0.82-1.58) | 0.450 | 0.96 (0.57-1.63) | 0.881 | 1.34 (0.80-2.26) | 0.250 |

BMI was categorized into underweight (<20.0 kg/m^2^), normal (20 to 24.9 kg/m^2^), overweight (25.0 to 29.9 kg/m^2^), and obese (≥30.0 kg/m^2^). Model was adjusted for age (continuous), sex (female or male), race/ethnicity (non-Hispanic white, non-Hispanic black, Mexican American, or other), education (less than high school, high school or equivalent, or college or above), smoking status (never smoker, former smoker, or current smoker), physical activity (inactive or active), anti-diabetic medications(no or yes), hypertension (no or yes), and cardiovascular disease (no or yes).

**Figure S1** Proportion of cause-specific deaths among participants aged ≥50 years with type 2 diabetes


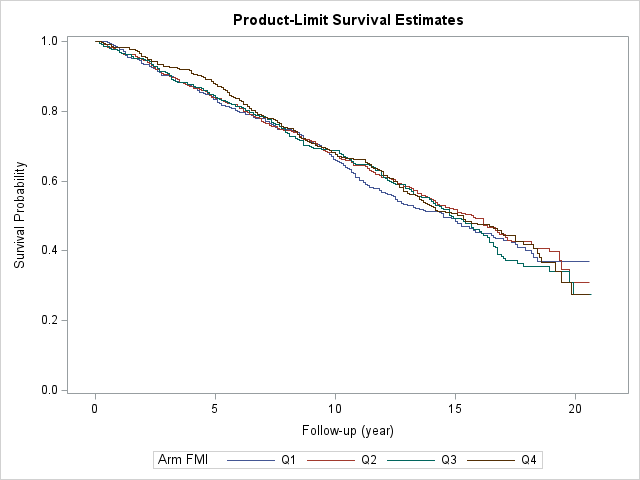

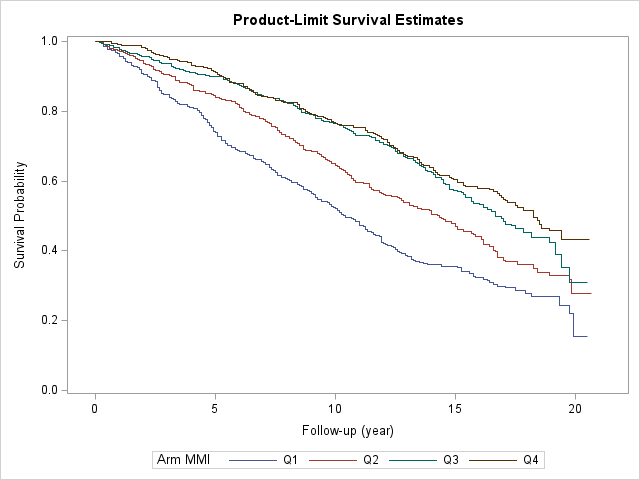


**Figure S2** Kaplan–Meier curves showing overall survival across arm fat mass index and arm muscle mass index

FMI, fat mass index; MMI, muscle mass index; Q1, first quartile; Q2, second quartile; Q3, third quartile; Q4, fourth quartile.


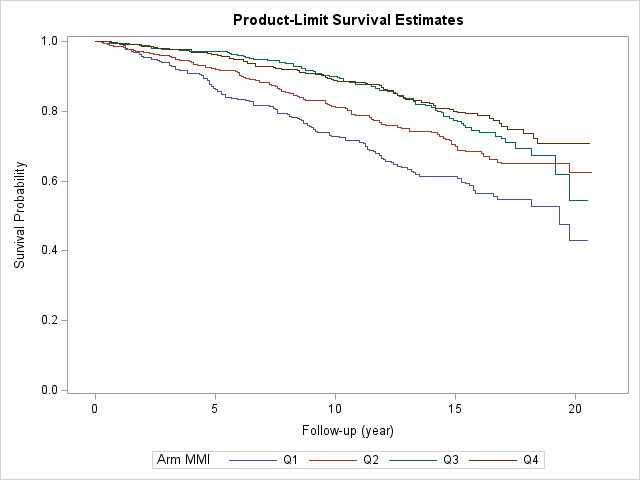


**Figure S3** Kaplan–Meier curves showing survival from CVD mortality across arm fat mass index and arm muscle mass index

FMI, fat mass index; MMI, muscle mass index; Q1, first quartile; Q2, second quartile; Q3, third quartile; Q4, fourth quartile.


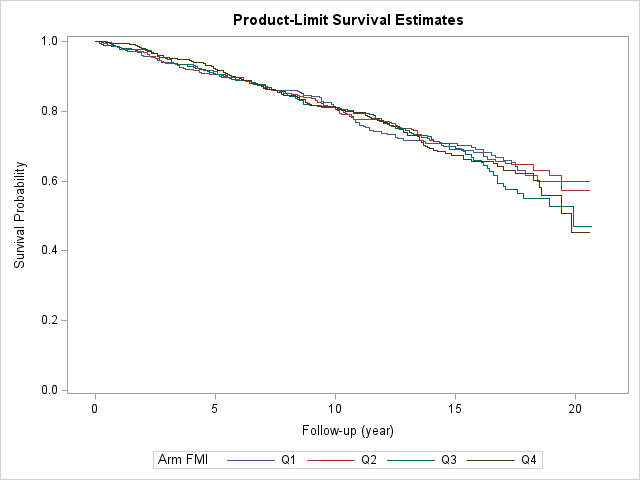


**Figure S4** Kaplan–Meier curves showing survival from non-CVD mortality across arm fat mass index and arm muscle mass index

FMI, fat mass index; MMI, muscle mass index; Q1, first quartile; Q2, second quartile; Q3, third quartile; Q4, fourth quartile.

**Text S1 Data collection for physical activity**

Participants were asked to report the intensity level (i.e., moderate- or vigorous-intensity), frequency, and duration of physical activity during the past 30 days. In addition, they were asked to report their engagement, frequency, and duration in daily activities (i.e., walking, bicycling, and tasks around home/yard) during the past 30 days. The NHANES guidelines suggested a metabolic equivalent (MET) score of 4.0 for one minute for walking or bicycling and a MET score of 4.5 for tasks around home/yard. These activities were thus defined as being of moderate-intensity (3.0 to 5.9 METs). Together, total minutes per week of moderate- and vigorous-intensity physical activity were calculated.

**Text S2 Calculations of body compositions**

FMI (kg/m^2^) = Whole-body fat mass (kg) / height^2^ (m^2^).

Trunk FMI (kg/m^2^) = Trunk fat mass (kg) / height^2^ (m^2^).

Appendicular FMI (kg/m^2^) = (Leg fat mass + arm fat mass) (kg) / height^2^ (m^2^).

Leg FMI (kg/m^2^) = Leg fat mass (kg) / height^2^ (m^2^).

Arm FMI (kg/m^2^) = Arm fat mass (kg) / height^2^ (m^2^).

MMI (kg/m^2^) = Whole-body muscle mass (kg) / height^2^ (m^2^).

Trunk MMI (kg/m^2^) = Trunk muscle mass (kg) / height^2^ (m^2^).

Appendicular MMI (kg/m^2^) = (Leg muscle mass + arm muscle mass) (kg) / height^2^ (m^2^).

Leg MMI (kg/m^2^) = Leg muscle mass (kg) / height^2^ (m^2^).

Arm MMI (kg/m^2^) = Arm muscle mass (kg) / height^2^ (m^2^).
